# Getting the Baby on a Schedule: Dutch and American Mothers’ Ethnotheories and the Establishment of Diurnal Rhythms in Early Infancy

**DOI:** 10.1002/cad.20336

**Published:** 2020-05-25

**Authors:** Saskia D. M. van Schaik, Caroline Mavridis, Sara Harkness, Margaretha De Looze, Marjolijn J. M. Blom, Charles M. Super

**Affiliations:** ^1^ Utrecht University; ^2^ University of Connecticut; ^3^ Utrecht University; ^4^ The Council for Health and Society

**Keywords:** American mothers, cultural differences, cultural models, diurnal rhythm, Dutch mothers, infants, parental beliefs, zeitgebers

## Abstract

One of the earliest challenges for infants and their parents is developing a diurnal sleep–wake cycle. Although the human biological rhythm is circadian by nature, its development varies across cultures, based in part on “*zeitgebers*” (German: literally “time‐givers”) or environmental cues. This study uses the developmental niche framework by Super and Harkness to address two different approaches to getting the baby on a schedule. 33 Dutch and 41 U.S. mothers were interviewed when their babies were 2 and 6 months old. A mixed‐methods analysis including counts of themes and practices as well as the examination of actual quotes shows that Dutch mothers emphasized the importance of regularity in the baby's daily life and mentioned practices to establish regular schedules for the baby's sleeping, eating, and time outside more than American mothers did. The U.S. mothers, in contrast, discussed regularity less often and when they did, they emphasized that their baby should develop his or her own schedule. Furthermore, actual daily schedules, based on time allocation diaries kept by the mothers, revealed greater regularity among the Dutch babies. Discussion focuses on how culture shapes the development of diurnal rhythms, with implications for “best practices” for infant care.

Newborn babies are, to a large extent, nocturnal creatures. Often born during the night, they seem most alert and bright‐eyed during the hours when their exhausted parents most long to be asleep. Fortunately, this early phase does not last very long for most babies, as their developing brains and bodies shift toward patterns of sleeping, eating, and activity typical of the mature human species. Depending on the sociocultural context, however, the establishment of regularity in such diurnal patterns may be more or less encouraged, and take place earlier or later in infancy. The variability in those patterns across cultures illustrates the plasticity of human behavioral development—babies are both creatures of nature and cultural beings from the beginning (Super & Harkness, 1982, 2010, 2013).

The development of the sleep–wake cycle is particularly important because it is a fundamental biological rhythm with which behavioral routines must be coordinated. Diurnal sleep patterns and, relatedly, activity of the hypothalamic–pituitary–adrenal axis, are regulated by an internal biological clock that is largely self‐sustaining; that is, the biological rhythm is expressed even when environmental conditions are not held constant (Aschoff & Pohl, 1978; Czeisler et al., 1999). Nevertheless, such biological rhythms, with maturity, become phase‐locked or entrained to regularly occurring environmental cues (Blumberg, Gall, & Todd, 2014). The human sleep–wake cycle, for example, typically mimics the cycle of night and day, such that arousal from sleep takes place at the beginning of the day and winding down to go to bed happens at night. A term commonly used for reliable environmental time cues that support the establishment and maintenance of biological rhythms in daily life is *zeitgeber* (from the German, meaning literally “time giver”). Although exposure to light has received the most attention as a zeitgeber (Goldman, 2001), other cues such as regularity in meals or social activities can also function to entrain biological rhythms (Blom, 2007; Richter et al., 2004; Stephan, 2002).

Humans are unique in that our environmental cues are largely socially constructed, and thus can vary quite dramatically across individuals or populations. The theoretical framework of the developmental niche (Super & Harkness, 1986, 1999) is useful for identifying and studying the infant's culturally structured environment for development; it highlights the physical and social settings of daily life, the customs and practices of care, and the psychology of the caretakers, especially parental ethnotheories (Harkness & Super, 1996; Harkness et al., 2007). This third component of the developmental niche plays a particularly crucial role in shaping the ways in which parents and other caretakers manage their infant's daily routines, as further specified in a heuristic model (Figure 2.1). At the top of the model are mostly implicit, linked ideas about children, parents, and the family; these include a broad array of abstract concepts, related, for example, to emotional closeness and the need for stimulation. Domain‐specific ideas are next in the model, and these beliefs are usually more readily articulated, concerning, for example, shyness, and expectations for school success. These more specific ideas are elaborated in beliefs about appropriate practices and their expected outcomes, for example, speaking gently, and promoting creativity and intellectual pleasure. Intervening factors (such as actual individual differences, external constraints on routines, or competing ideas about good parenting) mediate the relationship between ideas and actual practices. Developmental outcomes are thus seen in the model as the product of the practices, not the direct result of the beliefs. Following this model, the current paper aims to articulate the actual practices relating to the establishment of daily rhythms in infants through zeitgebers, as described by mothers. In short, we will link cultural models to the actual achievement of stable diurnal rhythms by elaborating a description of real‐life practices.

**Figure 2.1:**
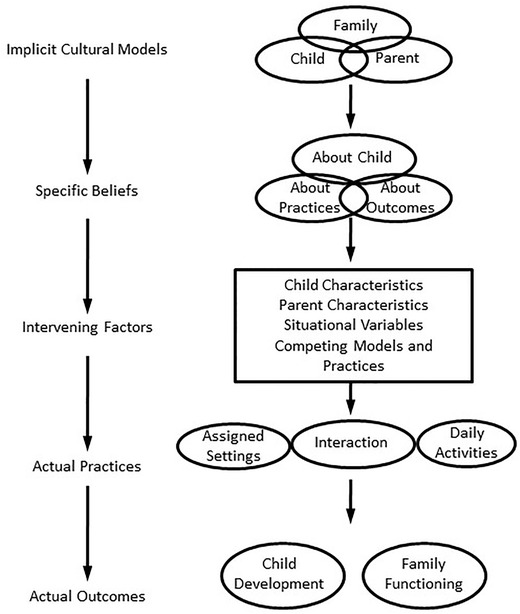
Theoretical model of Parental Ethnotheories, Practices, and Outcomes. *Source*: Adapted from Harkness, S., & Super, C. M. (2012). The cultural organization of children's environments. In L. C. Mayes & M. Lewis (Eds.), *The Cambridge handbook of environment in human development*, pp. 498–516. New York: Cambridge University Press.

In previous research, we identified the Dutch “three R's” of caretaking as a particularly well‐articulated parental ethnotheory related to the development of regularity in diurnal rhythms during infancy (Super et al., 1996). The “three R's”—*rust* (rest), *regelmaat* (regularity), and *reinheid* (cleanliness)—form a coherent set of guiding principles for baby care, and relatedly, dealing with ordinary problems of infant development such as infant colic, excessive night waking, or fussiness. The roots of this approach go back at least back to the influential seventeenth‐century Dutch physician Johan van Beverwijck, who devoted a full chapter to sleep in his classic work *The Treasure of Health* (van Beverwijck, 1649), and who was prescient in his approach to healthy daily routines and disease prevention. The “three R's” principle was codified as such by the end of the nineteenth century (van Hulst, 1905), and by 1907, some 15,000 copies of the parenting guide with that title had been distributed in the Netherlands (Rose, 1907). The concept is still widely espoused by both parents and professionals, as both rest and regularity are often mentioned in documents published by the national well‐baby clinics (Kesler et al., 2018; LaHaye et al., 2013), and in Dutch pediatric research (Blom, van Sleuwen, de Vries, Engelberts, & L'Hoir, 2009).

Research on the Dutch “three R's,” carried out in the 1990s, compared this cultural model with ideas about the development of sleep patterns in the United States, finding that although parents in both samples expressed concern about their child's sleep patterns, they differentially emphasized particular themes. For the Dutch parents, the two dominant themes were that parental maintenance or imposition of regularity, particularly regarding sleep, is very important, and that failure to do so in the early months or years of life would have unfortunate sequelae later on. In contrast, when the American parents discussed rest and arousal, the most frequent theme was to the effect that some children are naturally or innately more or less regular in their sleeping or other activities than others. Actual patterns of sleep varied quite dramatically between the two samples, with the Dutch babies averaging 2 hours more sleep per day, on a more regular schedule, at 6 months, with diminishing but still significant differences up to age 4 years (Super et al., 1996). Relatedly, the evening meal was found to be more regular for the Dutch children, both at the individual and group levels (Super & Harkness, 1999).

A more recent comparison of cultural themes and related practices of parenting expressed in interviews by mothers of 2‐month‐old infants in Italy, Korea, the Netherlands, Spain, and the United States showed, likewise, that regularity of routines was a far more salient concern for the Dutch mothers than it was for any other group (Harkness et al., 2007). The theme of regularity in the discourse of Dutch mothers, moreover, was found to be linked to other themes having to do with the development of emotional and behavioral self‐regulation, and implicitly with cultural concepts of both development and the family. Middle‐class US parents in the study, in contrast, expressed little concern with establishing regular schedules for their babies as a goal in and of itself; rather, these parents talked most about the importance of providing stimulation to support the baby's optimal early development.

Building upon this previous research, the present study provides new evidence on the links between parents’ ethnotheories regarding the development of diurnal rhythms in early infancy, and the practices they use to instantiate these ideas, with particular reference to four aspects of daily routines that can function as social zeitgebers: sleeping, eating, time outside (and therefore exposure to natural daylight), and particular activities. Further, the present study analyses whether these differences in practices correspond to actual observed occurrences of regularity in these four aspects of infants’ daily routines.

## Methods

### Participants

Data presented here were drawn from the International Baby Study, a longitudinal study of parental ethnotheories, practices, and the development of self‐regulation and reactivity from the prenatal period through the first 2 years of life. Participants were drawn from two middle‐class populations in the Netherlands and the United States between 2001 and 2005. It is important to keep in mind that our samples here are located in particular communities within their respective countries. Our purpose was not to recruit nationally representative samples; we are studying parents’ cultural models and parent behavior, in a particular time and place. The question of how generalizable our results might be to other populations is beyond the scope of the present research, although some insight can be gained from comparing results here to other studies.

The Dutch sample was recruited from towns located in the most densely populated area of the Netherlands, south of Amsterdam. All participating families lived in residential communities in attached row houses, apartments, or their own freestanding homes, with pedestrian walkways and bike paths along the streets providing easy access to schools, playgrounds, small parks, and village shopping. Almost all the families had extended family living nearby, and regular help with childcare was a common feature of family life. Many of the mothers later returned to employment, often part time, in such positions as teacher, nurse, sales clerk, or accountant. Fathers in the Dutch families were employed in a range of occupations, including store manager, chemist, salesman, teacher, landscaper, and journalist.

The U.S. families lived in suburbs and small towns in central and eastern Connecticut. Almost all owned or rented their own freestanding homes, which were usually surrounded by a small yard. Although this arrangement provided easy access to the outdoors and open play space, families generally had to drive to shops, schools, or friends’ houses. Few mothers had family in the same neighborhood, although most had relatives in the same or a nearby town. Most of the mothers in this sample were employed before giving birth, and the majority had resumed work, at least part time, by the baby's second month, as paid maternity leave was limited. The mothers’ occupations covered a wide range including salesclerk, foster parent, social worker, attorney, librarian, and preschool teacher. The fathers, too, worked in varied occupations, including truck driver, professor, attorney, software technician, and business manager. The sampling criteria were as follows: both parents were native‐born; the first language for both the mother and father was the dominant language of the community (English or Dutch); one or both parents worked; and there were no serious health problems for either the mother or infant. Other demographic characteristics such as parental age, education, employment, and childcare arrangements, were allowed to vary naturally as these tend to be integral aspects of different cultural systems.

### Procedures

Data collection including background information questionnaires, in‐depth interviews, and daily diaries, was carried out in families’ homes, at times convenient to them, by trained graduate students or the principal investigators. Before collecting this data, researchers paid an initial visit to the home to describe the study, obtain formal consent, and explain the materials left for completion before the second visit and interview. Most interviews were done solely with mothers due to partners’ work schedules, although fathers’ responses were incorporated when they were present and wished to take part.

The interviews were tape‐recorded and transcribed in the original language for analysis by native speakers. Translations were used only for purposes of communication between researchers of each site and for quoted examples in publications. This process was facilitated by the fact that three of the present authors have good knowledge of both languages.

### Measures

#### Family Background

A brief questionnaire was used to collect information on family background, including age, education, number of other children, employment type, family of origin, plans for returning to work, and religious background. The questionnaires were reviewed and collected at the first visit after obtaining consent.

#### Interviews, Coding, and Reliability

Interviews were designed to elicit detailed discussions of parental ethnotheories and related customs of care, particularly with regard to the organization of infants’ daily routines. Thus, parents were asked to narrate a typical day with the baby, which provided insight into overall family life, and in particular patterns of regularity in various activities and caregiving practices (e.g., putting the baby to bed). Parents were also asked directly about their goals and strategies for establishing regular schedules of sleep and feeding. Additionally, the interview included more general questions about important things to do for the baby, and what he or she seemed to need at the present age. Thus, parents had several opportunities to discuss issues of regularity, in response to specific questions as well as spontaneously as something they valued.

The coding scheme was broadly structured by themes that emerged in our earlier analysis of ethnotheories (Harkness et al., 2007), and an examination of zeitgebers in these two samples based on the animal and human literature cited earlier (Blom, 2007). The focal areas for coding were sleeping, eating, exposure to daylight, other activities, and a general category for cases in which parents discussed regularity but not in relation to a specific aspect of daily life. For each topical area, codes were constructed for both themes and related practices. In all, there were 62 such codes, and the total of their occurrences was used as the denominator in calculating percents (described below). An additional 12 codes focused on how parents conceptualized or delineated the baby's daily schedule for the four specific domains (sleeping, and so forth): by reference to an exact “clock” time, by a general time of day (e.g., “in the morning”), or in relation to other events as cues (e.g., “when his father comes home”).

The coding list was developed inductively, in multiple iterations, through close readings of interviews. Following the general principles of thematic analysis (McClelland, 1976), an initial list was tested upon multiple interviews, discussed among coders and investigators, and revised until it encompassed all relevant concepts of regularity. Final coding was done using N‐Vivo Version 1.3 (QSR, 2002), a commercial software program for qualitative analysis, at the level of sentences and brief passages. In order to establish inter‐coder reliability, three interviews with U.S. mothers were coded by three coders independently. U.S. interviews were used since English was the common language for all the coders. Reliability was established at 80% or above.

#### Diaries

We asked the parents to keep a dairy of the baby's activities for three consecutive days prior to the second home visit and interview, in order to obtain an empirical estimate of the actual use of zeitgebers and associated differences in sleep behavior. The diary form we provided contained lined rows to be used for separate time segments, and columns to enter the infant's activity (including sleep and feeding), as well as location and position (e.g., lying in playpen in living room, sitting in stroller in the park), identity of the people present, and the time of day at which any of these variables changed (hence, beginning a new line). Parents were requested to keep the diary for three consecutive days (but not including holidays or atypical days). In virtually all cases the diaries were completed by the mother.

### Data Transformations and Analysis

#### Interviews

Frequency counts for the use of each code (i.e., number of passages coded) were calculated for each interview. In order to control for individual differences in interview length, we transformed each frequency count to its percentage of all coded passages (i.e., 100 times the count for each code divided by the total count). Analysis of variance (ANOVA) indicated that there was no systematic difference between the Dutch and U.S. samples with regard to the number of codes per interview (M_NL_ = 36.5 and M_US_ = 42.0).

Two‐way Analyses of Variance (Age Group × Culture) were used to analyze continuous variables approximating a normal distribution; when assumptions of normality were not satisfied, the scores were ranked and then normalized prior to analysis. For codes that were mentioned by less than 50% of the group at 2 or 6 months, dichotomous variables were created, such that a score of 1 indicated the idea had been mentioned at least once, and 0 indicated no mention in that interview. Group and age differences on these dichotomized variables were tested using chi‐square tests. When conceptually appropriate, single codes within a domain (for instance, all topics of coding for “clock” references) were summed into larger, composite variables. In order to simplify the presentation of results, we omit intermediate statistics such as *F*, *χ*², and their related degrees of freedom, and report instead a *p* value and effect size: partial eta squared (*η* ^2^) for Analyses of Variance and phi (*φ*) for chi‐squared analysis.

#### Diaries

Many of the dairies were not completed for three full 24‐hour days, but we could reliably extract 2 consecutive days (48 hours) starting at 7 AM. The diaries were converted to a computer database consisting of variables representing key elements (e.g., sleep/wake, feeding/not) for sequential 1‐minute intervals. Using this database of 2,880 consecutive intervals per child, we calculated regularity measures for time outside, sleeping, and feeding. With regard to the first two, for every segment in the first day (segments 1—1,440) marked as outside (or sleep), we examined the corresponding status in the second day (segments 1,441–2,880), and calculated the percent also outside (or sleeping). To balance for asymmetry, we repeated this process in the other direction (day 2 to day 1), and took the average. The resulting metrics indicate the percent of segments the infant was outside (or asleep) on either day for which the infant was also outside (or asleep) on same 1‐minute segment on the other day—in short, the temporal regularity of being outside and of being asleep.

We found that the diaries did not reliably indicate the conclusion of feeding (breast or bottle), as the baby might fall asleep in the mother's arms and remain there for some time, with the mother recording only the time she ended the process, for example, by putting the baby down in bed. For measuring regularity in feeding, therefore, we used only the number of feeding sessions, calculating two measures: the absolute difference in number across the 2 days, and the day‐to‐day correlation.

## Results

Demographic information for the Dutch and U.S. samples is shown in Table 2.1. The gender balance of infants is approximately the same in both samples, although more babies in the U.S. sample were firstborns. Mothers and fathers in both groups averaged about the same age. U.S. parents had about two more years of education than their Dutch counterparts, although fathers’ occupational status, as indexed by Hollingshead's scale (Hollingshead, 1975), was similar. Of mothers who were employed prior to giving birth, the U.S. mothers were considerably more likely to be working full time. Three‐fourths of the Dutch mothers and half of the U.S. mothers planned to return to work; the Dutch mothers, however, planned to return when the baby was about 12 weeks old, whereas the U.S. mothers planned a return around 6 weeks. It is likely that this difference reflects differences in Dutch and U.S. family leave policies as well as other features of the larger environment.

**Table 2.1:** Demographic Characteristics

	Value			
Variable	NL	US	Statistic	*p*
*n* at 2 months	19	21		
*n* at 6 months	14	20		
Child's age at 2‐month interview (median weeks)	8.9	9.4	*z* = 1.31	.19
Child's age at 6‐month interview (median weeks)	28.0	26.4	*z* = −1.26	.21
Child sex (% male)	63	76	*χ*²(1) = 0.81	ns
Child birth order (% first)	15	52	*χ*²(1) = 6.37	.01
Mother's age (mean years)	32.5	31.5	*t*(34) = 0.76	ns
Father's age (mean years)	35.9	35.1	*t*(34) = 0.68	ns
Mother's education (median years)	14.0	16.0	*z* = 2.37	.02
Father's education (median years)	14.0	16.0	*z* = 1.74	.08
Father's Hollingshead Occupation Score (median)	3.0	3.0	*z* = 0.05	ns
Mothers employed, prior to birth (%)			*χ*²(2) = 16.86	.0002
Full (≥ 35 hours per week)	11	50		
Part	89	25		
Not	0	25		
Mother's work prior to birth (mean hours per week)	22.5	39.0	*t*(32) = −4.21	<.0001
Mothers’ planned return to work (median weeks)	12.0	6.0	*z* = 2.32	.02
Mothers employed at 6 months (%)			*χ*²(2) = 2.47	.29
Full time	0	15		
Part time	71	55		
No	29	30		

In light of the significant sample differences in maternal education, employment status, and child's birth order, all the analyses presented here were re‐examined for possible confounding influences. Applying regression controls within samples, none of the background variables were correlated with any of the interview variables. In the most demanding model—controlling for maternal confounds in the pooled sample prior to the main effects of sample and age, a procedure that biases against finding sample differences—the regression controls were rarely significant and in no case did they alter the conclusions presented here.

Following the conceptual model of parental ethnotheories and practices described in the introduction, we first consider mothers’ general ideas about regularity in relation to babies’ daily routines. Did the Dutch and U.S. mothers consider a regular schedule for babies important—and if so, why and for what purposes? Relatedly, what evidence did mothers provide of their own awareness of time as an organizer of their baby's daily routines? We then take a closer look at mothers’ talk about how they viewed regularity in the specific zeitgebers of sleeping, eating, time outside, and particular activities, and how these ideas were instantiated in reported practices.

### Implicit Cultural Models: The Importance of Regularity (or not) in Babies’ Daily Routines

According to the Dutch mothers in our study, regularity of routines was important both in itself, and in relation to other themes of parenting. A composite summary index totaled each mother's comments on each of the following topics, as a percent of the total for all 62 primary themes: (a) the importance of the baby adapting to the family's schedule; (b) the importance of the baby adjusting to an upcoming daycare schedule; (c) the importance of regularity; (d) planning the schedule in general; and (e) discussions of a high degree of regularity actually occurring in the baby's daily life. The results of a two‐way ANOVA indicate a significant sample effect of a medium size (Cohen, 1977): *p* = .002, *η* ^2^ = .18, with no age or interaction effects. Dutch mothers made many more statements about the overall importance of regularity than did their U.S. counterparts, M_NL_ = 8.4, M_US_ = 1.8. As one Dutch mother of a 2‐month‐old stated, “I think regularity is important for children, and actually also for yourself.” As one of the cornerstones of the traditional “three R's” of Dutch childrearing, regularity was implicitly linked to its other main component, rest, by the mother of a 6‐month‐old who commented, “I do like to stick to a schedule; just because, well yeah because of her rest too!” Another mother of a 6‐month‐old evoked a whole array of related ideas about regularity, rest, emotional security, love, and the needs of the family:

I: What do you think is the most important thing you can do now for Noah?

M: Love, affection. Just like the three R's—rest, regularity, and cleanliness.

I: Ya, why do you think so?

M: Well, we already have an organized family. One has to go to school and one to kindergarten. He already is in a schedule and it is very structured.

I: But do you think that is important?

M: Yes I do think it is important. So they know where they stand. And, next to that, just love of course.

Whereas Dutch mothers stressed the general importance of regularity in the baby's daily life, U.S. mothers described a rather different approach. In a few cases, U.S. mothers actually were explicit in stating that regularity was *not* an important aspect of the baby's early development. As one mother of a 2‐month‐old explained, “I think kind of not being on a schedule has been good for him in a way, just kind of being able to go slow, and if we need to go in the car we go in the car, and it's not a big fuss and it's not a big to‐do, we just do it.” Nearly a third of the U.S. mothers expressed a similar sentiment at 2 months, six times the rate in the Netherlands (*p* = .05, *φ* = .31).

The idea of a regular schedule seemed novel to some of the U.S. mothers: as one mother of a 2‐month‐old commented, “Right now, he's pretty much setting the schedule. I'm just starting to now really pay attention to his schedule actually, since I started doing this [research].” The perception that the schedule was set by the baby was widely shared among the U.S. mothers at both age points: as a mother of a 6‐month‐old said, “She calls the shots and we work around it basically.” Overall, the average percent of comments to this effect was significantly higher in the United States, M_NL_ = 35%, M_US_ = 66%, *p* < .001, *η* ^2^ = .32. We did not find an effect of age or an interaction effect.

The importance of a regular schedule was often explained by the Dutch mothers in terms of the need for the baby to adjust to the schedules of the rest of the family, as well as to the schedule of daycare for those babies who would soon be attending (although usually part time). One mother of a 2‐month‐old asserted the first assumption clearly: “No, he basically just has to join our schedule—too bad for him!” Far fewer mothers in the U.S. sample felt that the baby should be expected to adjust to the family's routine, NL = 50%, U.S. = 18%, *p* = .007, *φ* = .34. When this sentiment was expressed in the United States, it was more in terms of “hoping” the baby would get into a regular pattern; however, as illustrated by this mother of a 2‐month‐old, it was not expected that one can impose much of a routine on such a young infant:

I'm hoping I can keep him, you know, help him to get more of a schedule and he's still young so it's basically what he wants when he wants it to some degree but I hope to, ‘cause I think that that's… you know we're pretty routine in this house as far as what time we would do things, and everything is pretty much done the same time every night unless something comes up or we're out, so I think that that's pretty important especially when they start getting older.

In keeping with cultural differences in ideas about the importance of regularity and the need for the baby to adapt to externally imposed schedules, we found substantial differences in the extent to which the Dutch and U.S. mothers referred to time. The Dutch mothers were more likely to refer to clock times, M_NL_ = 18%, M_US_ = 11%, *p* < .0002, *η* ^2^ = .18. Overall, references to clock time was more common among mothers of the older infants (M_2mos_ = 12%, M_6mos_ = 16%, *p* < .003, *η* ^2^ = .07); but the interaction of Age and Sample was not significant. Dutch mothers also referred more frequently to broader parts of the day than did the U.S. mothers, M_NL_ = 11%, M_US_ = 6%, *p* < .0009, *η* ^2^ = .15. No effect of age or interaction effect was found for this measure. As one Dutch mother described her 6‐month‐old's day,

Well, generally speaking, it is…they are pretty set times. When he wakes up at eight, I can just put him back to bed at ten, half past ten and in the afternoon usually it's about one.

In contrast, when the U.S. mothers did make reference to the clock, they tended to be less precise, often naming a time range *between* which they could expect the baby to eat, sleep, or do other activities. Further, they often emphasized the contingent nature of the schedule on any particular day. As the U.S. mother of a 6‐month‐old described her baby's patterns:

Uh, he has his own room, in his crib, and he sleeps…I'm working on trying to get him into bed between 8 and 9, which works most of the time, and he will sleep through the night and wake up anywhere in between 5:30 and 7, and uh, it just depends what time he gets down, and then he'll wake up…

Further, the U.S. mothers were more likely to refer to event cues (such as returning home from shopping) than were the Dutch mothers: M_NL_ = 6%, M_US_ = 9%, *p* < .05, *η* ^2^ = .05. Again, no age effect or interaction effect was found.

### The Cultural Construction of Zeitgebers: Ideas About Sleeping, Eating, Time Outside, and Activities

For babies in both samples, daily routines were constructed around core activities including sleeping, eating, time outside, and particular activities. Insofar as mothers used these to establish a regular schedule, they functioned as culturally constructed zeitgebers. As a closer look at each of these domains indicates, cultural differences between the two groups both reflect the general themes already seen, and provide further insight into how ethnotheories regarding regularity were instantiated.

#### Sleeping

In order to get a broad sense of the salience of regularity in sleeping patterns, we made use of a summary index that combined all sleep‐related ideas and practices (as a percent of all 62 codes). A two‐way ANOVA indicates that Dutch mothers made a significantly higher percentage of comments related to regularity in sleeping patterns than did U.S. mothers, M_NL_ = 68%, M_US_ = 51%, *p* < .001, *η* ^2^ = .17, with significantly more such comments in both samples at the older age. Although the Dutch mothers expressed a strong conviction that a regular sleep schedule was necessary, some commented that the baby did not necessarily like following a routine. As one Dutch mother of a 6‐month‐old explained:

M: Well, she doesn't really like the thirty minute nap at the end of the [afternoon]; it usually is quite tough for a while.

I: Yes, but it's necessary?

M: Well, yes, otherwise she won't make it till 7 o'clock [bedtime].

According to these mothers, keeping a regular schedule for sleeping was important in order to avoid the baby's being “confused” by changes. In addition, some Dutch mothers also expressed a concern for their own needs. As one mother of a 2‐month‐old recounted:

Yes, that with her, she was always awake from 7 till 11 or so; she was awake. And at a certain point I was like, ya, we don't have any time for yourself anymore. She's just always awake. She just, at a certain point, she just has to go to bed at around half past 6 or half past 7. …And so then we started giving her a bath in the evening, and that, let's say 15 minutes earlier per week, and so eventually she went to bed at half past 7.

In contrast to the Dutch mothers, the U.S. mothers’ discourse was characterized by their discussions of how they “hoped” to establish a regular sleeping pattern, but nonetheless felt that they had to accept the baby's own demands. For example, one mother, at 2 months, summarized her view of getting the baby to sleep on a schedule:

But see, you can take a horse to water but you can't make him drink. You can't make a baby sleep either.

Another U.S. mother, also at 2 months, responded to the question of how she thought her baby's schedule might develop over time:

What I'm hoping…(laughing), I hope that it gets more regular, you know, that I can predict, you know, exactly what time you [the researcher] can come over and she'll be up. Um, and you know, her nights are still not absolutely consistent, although I've seen a routine kicking in, and so I know kind of around what time she'll wake up, and what time I put her back in for a nap, and what time she'd have a feeding, so hopefully as maybe the next month or two goes on, that'll be more regular.

At 6 months, in response to the same question, it is clear that this mother's “hope” of regularity had not yet been fully realized, but she described her baby as an active agent who was “figuring out” her schedule” and “working” toward a more regular routine:

Um, hopefully it will just get more predictable with her feedings and her sleeping. Like I said, like the mornings are, you know, very routine and predictable. Um, but then after that first nap…I kind of feel like I play a guessing game as the day goes on. So I'm hoping that with her schedule it just gets more predictable and I, you know, her naps get more regular… and I think that that is hard because having, you know, a three‐year‐old and his schedule is so predictable… that I keep feeling like hers should be that way too, and that I've got to remember you know she is working on it, and she is going to have growth spurts, and she is figuring out her sleeping schedule, and so little by little I hope that gets better.

Consistent with this approach, and again in contrast to the Dutch mothers, the U.S. mothers were also more likely to discuss how they adjusted their own daily activities such as work, errands, or even getting a little rest themselves, in order to accommodate the baby's observed sleep patterns. As one mother of a 6‐month‐old laughingly commented,

[Be]cause that's kind of the rule in the house, you can take a nap but only when the baby is napping…You're up when the baby wakes up.

For some of these mothers, adapting to the baby's naptime was seen as a helpful strategy for accomplishing their own work, given that a regular naptime could not be taken for granted. As the mother of a 2‐month‐old explained,

When he sleeps…I can go get housework done or go get work done. So I'd rather do that than like force him to get back up and then [a] chance of him not sleeping when I need to work. So, I'm just kinda going with the flow a little bit.

For both the Dutch and U.S. mothers, soothing practices such as playing soft music, talking, singing, reading, and caressing the baby were mentioned as helpful for getting the baby to establish a regular sleep pattern. The Dutch mothers, however, more frequently described using these practices, particularly at 6 months, M_NL_ = 3%, M_US_ = 1%, *p* < .02, *η* ^2^ = .07. One Dutch mother of a 6‐month‐old elaborated on her routine in tucking the baby into bed:

I usually first change him into his pajamas, then I'll sing a bit and hold him and cuddle him and he has a kind of wooden doll with a feather hanging from the ceiling over his bed and…if you pull it a bit then…well he just loves that! So then he will look at me in this way, almost like he's sort of following it with me. And a little music box; those are the things I basically always…always do. And at someone else's house, I will at least always gently put his pajamas on, cuddle a bit and sing a song.

A sleep‐promoting practice mentioned by nearly a third of the U.S. mothers (29%) but only 1 Dutch mother, was that of taking the baby into their own bed (*p* = .003, *φ* = .34). As a U.S. mother of a 2‐month‐old described:

Um…because he was fussing, what I will do sometimes is I will nurse him, I will change him, and if he is still fussing, I will go to bed with him and I will lie down beside him, and usually within ten minutes he will be out like a light; and so I was dead tired so he slept in the bed with me, and when my husband came to bed he put him in his crib and then he slept. So that was a fairly typical day.

#### Eating

As with the topic of sleep, we began our analysis of regularity and feeding practices by examining group differences in the overall salience of this theme. A two‐way ANOVA, using a composite summary index of all codes for eating discussion and practices (as a percent of all 62 codes), showed no Sample, Age or Sample × Age interactions. Further analyses of specific ideas and practices related to feeding, however, did reveal some distinct sample differences. As with the topic of sleep, more Dutch mothers (at 2 months) spoke of the need for the baby's feeding schedule to be coordinated around the needs and routines of other family members, NL = 26%, U.S. = 5%, *p* = .06, *φ* = .30. For example, in response to the interviewer's question about the baby's feeding schedule, a Dutch mother responded,

…that I do something different especially for her? Well, yes, I just give her, look, I have my own routine and she just follows that. So when I drink my coffee, I'll grab her a fruit snack, and in the afternoon when I drink something, she'll get the bottle and it all just works like that and sometimes something might change, but we usually have the same daily routine.

Once again, in contrast to the Dutch mothers, the U.S. mothers were more likely to discuss the ways in which they or the family adapted to what they perceived to be the baby's own emerging pattern. For example, one mother, at 2 months, responded to the interviewer's question about whether she scheduled the baby's feedings:

Nope. It's on demand. When he screams, we provide.

Likewise, a U.S. mother of a 6‐month‐old baby recounted her attempts to schedule feedings, ultimately without success:

Yeah, it's pretty much all about Carly (laugh)…whereas like at the beginning I wanted it to be all about me and my schedule and Caleb's schedule and what needed to be done, and you know I was trying to get our schedule, you know, and then hers would fit in there. Um, with her and her personality and you know…I talked to the doctor about it and he said, “It's your will against hers.” Um, it's gotta be all about her. Which at the beginning with my prior interviews I always, I was gonna schedule her, I was gonna have her on this schedule, but it just is miserable if she is not doing what she needs to do, and that is her personality or that is the way she is now, so I kind of had to rearrange my plan…I make sure she gets enough food in her, but when she needs it and wants it.

Another intriguing sample difference emerged in relation to feeding: at both 2 and 6 months, the Dutch mothers talked about creating a regular feeding schedule by “stretching” intervals between feedings, an idea that was never mentioned by the U.S. mothers, NL = 24%, U.S. = 0%, *p* < .001, *φ* = .39. The practice of “stretching feedings” (*voedingen oprekken*) as described by the Dutch mothers involves creating a space of 3–4 hours between bottle feedings or nursing bouts. The Dutch parents in the following quote elaborate on how and why they implement clear intervals between the feedings of their 2‐month‐old baby:

F: Yes, so you do keep it [stretching feedings] in mind! If she starts crying two hours afterwards [after a feeding] you'll say: this can't be hunger and then you…you distract her to do something else to sort of force some kind of regularity.

M; Well, force…But it is true…because of my profession I know…

F: Yes, after two hours…

M: That…that after two hours she's digesting her food, it starts working and after four hours her food will be gone. So if you keep feeding after two hours her system just keeps on going.

F: Keeps on going.

M: Keeps on going and you have to give them rest too, so that's why I take that into account, not forcing, but just to help my child!

Another Dutch mother, also at 2 months, explained how “stretching feedings” was a successful strategy for helping her baby to be calmer:

And in her case, that was if I stretched the times between the feedings a bit, that in the end, it all went much better. That she slept through the night more easily. It seemed as if the more I fed her the more restless she became, whereas if I tried to stretch between the feedings, she was much calmer, so at some point I started stretching between the feedings during the daytime up to four hours.

Thus, the Dutch mothers expected—and apparently achieved—a regular feeding schedule for their babies as early as 2 months of age, in contrast to the U.S. mothers who evidently felt (and were supported in this by their own sources of expert advice) that even at 6 months, babies could not be forced into a regular feeding schedule that they might not be ready for.

#### Time Outside

Spending time outside at regularly scheduled times each day is an important zeitgeber, insofar as exposure to light provides a primary cue for the establishment of circadian rhythms (Aschoff & Pohl, 1978). The Dutch mothers in our study shared a traditional cultural belief that fresh air is important for health in general, and especially for infants and children. This belief, found in societies across northern Europe, is expressed in a variety of practices including putting babies outside (warmly covered, in their baby carriages) for naps even during the cold winter months. The idea of regularity in time outside is an added dimension that we found in our analysis of the Dutch mothers’ talk about taking their babies outside. Consistent with our findings related to sleep, the Dutch mothers made more, and more elaborated, statements about providing the baby with regular time outside than did the U.S. mothers, M_NL_ = 22%, M_US_ = 14%, *p* < .001, *η* ^2^ = .18, and they were more likely to refer to a time for going outside, marked by the clock, by a general time of day, and/or by events (the percent of mothers mentioning clock time at 2 months: NL = 68%, U.S. = 5%, *p* < .001, *φ* = .67). The importance of time in the “fresh air” for the baby, both awake and asleep, was described by the following Dutch mother of a 6‐month‐old:

It's so nice to have him outside a lot. And at home, well yes, like now it's a bit too chilly I think, so I'll keep him inside for a while, but when I sit outside, then he'll sit comfortably outside as well. And usually after the walk…when you've walked with the baby carriage and he's nicely sleeping in it, then I usually keep him outside and asleep. …Yes, just to be nicely outside…fresh outdoor air it's good for him! (Laughter). Then I'd actually rather keep him outside with an extra blanket…than take him inside with the stroller in the living room.

A conversation between a Dutch researcher and the mother of a 2‐month‐old also illustrates this shared cultural model:

M: Well, I definitely think that he needs to be “aired out” [*uitgelucht*]. And then usually we go to the shop, or the mailbox, or you just walk around a little. So he always comes along…There always is something that has to be done.

I: So you think it's important that he gets a “fresh nose” [*frisse neus*]?

M: Yes, or I'll just put him outside in the stroller for a while. Of course, it doesn't matter whether you're walking or he just stays outside.

As in the case of feeding, not only was regular time outside discussed in terms of its own inherent importance, but also in relation to rest and sleep. The following quote from the mother of a 2‐month‐old evokes the cultural model of the “three R's” and also makes explicit its connection with actual growth:

Well, yes, he is calm outside, so I like that [laughs], and I think that if children have a lot of rest and are calm, that they grow better.

In contrast to the Dutch mothers, some of the U.S. mothers of 2‐month‐old babies (but not at 6 months) talked about the absence of regularity in spending time outside, an occurrence that was only rarely mentioned by the Dutch mothers, NL = 0%, U.S. = 33%, *p* = .006, *φ* = .44. As the following U.S. mother of a 2‐month‐old recounted:

Um, we went outside Thanksgiving Day because it was such a beautiful day. We were outside quite a bit, but just um, regularly, usually in the past week, no. It has just been to the car and back. No, we've been running some errands here and there but, and that's so it's just been, she's been in her car seat and then from the house to the car, from the car to the store, or something like that. But we haven't spent regular time outside.

Although some of the U.S. mothers stated that spending time outside with the baby was important, they also mentioned various deterrents that kept them from doing so, including the lack of “places to go” for outdoor activities, and the weather they saw as either too hot or too cold. One mother of a 6‐month‐old who had recently moved to a nice new house set in the woods lamented,

I'm really, really lucky about that [the new house], but um…I mean I'm really isolated. I go…I haven't even walked out of the house today except to shake the rug out.

And to the interviewer's sympathetic “Oh, no,” the mother concluded, “Yeah, so…that's normal.”

#### Activities

Mothers often talked about other specific activities or events as markers in the infants’ day, including play and social interaction, physical exercises (e.g., “tummy time”), and activities geared toward cognitive enrichment, such as watching a video targeted at baby development, for example “Baby Einstein.” A two‐way ANOVA of the summary index for activities and events as zeitgebers (all time descriptors for activities and events, such as changes in caregivers, coordination of the baby's activities with those of the family, general statements on the importance of regularity in activities, as well as the actual regularity of their occurrence) indicates a sample effect only, with the U.S. mothers more likely to mention regularly occurring activities throughout the baby's day than the Dutch mothers (M_NL_ = 9.6, M_US_ = 19.0, *p* < .0001, *η* ^2^ = .17). Time for physical closeness was frequently mentioned by the U.S. mothers. For example, one mother of a 2‐month‐old described having the baby in bed with both parents while they watched TV after dinner. Another, at 6 months, described this routine following meals:

What I try to do every day is at meal times I'll have him…like I'll eat, he's eaten already, I'll eat and then I'll have him sit on my lap and we'll play at the table. You know, I just want the body contact.

The U.S. mothers also mentioned individual activities geared toward developing motor or cognitive skills, for example, bouncing in a “jumper” that exercised the baby's legs and also provided some “independent play time,” as one mother of a 6‐month‐old phrased it.

The Dutch mothers, who were less likely to discuss regularly occurring activities, nevertheless did mention activities focused on social interaction and physical closeness. There was little mention, however, of activities geared specifically toward development, such as motor development or learning to self‐entertain. The primary exception is “baby swimming,” which 86% of them discussed when the child was 6 months of age (compared to 25% of the U.S. mothers, *p* = .0005, *φ* = .60; at 2 months, the percentages are 37% and 20%, *p* = .21, *φ* = .20). Even in those instances, though, their developmental importance was downplayed in favor of simple social interaction. As one Dutch mother of a 2‐month‐old put it,

Yes! Yes…just nice and easy on my lap. Like I just had him here, on my knees, I'll lay him down…He likes…Yes, I just want a little bit of interaction. I don't really think…It's not a baby massage or baby swimming or anything like that.

It is noteworthy that as early as 2 months, a higher proportion of the U.S. mothers described efforts to coordinate the infant's activities with those of the rest of the family, NL = 5%, U.S. = 33%, *p* = .03, *φ* = .35. This is in contrast to U.S. mothers’ beliefs and customs regarding sleep and feeding. That is, the U.S. mothers tended to leave the timing of the sleep and feeding routines “up to” the unfolding pattern they saw in the baby, rather than attempting to impose a great degree of structure. In terms of activities, however, the U.S. mothers tended to stress the importance of working the baby into family activities, with the added goal of creating some time for the mother to attend to her own needs. As one mother of a 6‐month‐old recounted:

I mean, actually two main things that she does every day that she should probably be really used to is she watches a video of Baby Mozart and Baby Bach. Um, and she does one in the morning a half an hour so I can have my time, and I go on the treadmill and so I know she is in her swing, and she is able to watch that and she loves that. I mean once it pops on she is just giggling and smiling and um, and it's…I've come to recognize I think she is expecting it… And then also in the evening time when I'm making dinner, she watches it again or another video. Um, and it is on her own time and you know, so I think she really enjoys that, and it's good for me too.

#### Regularity Outcomes

Analysis of the diaries indicates more regular exposure to *zeitgebers* in the Dutch sample (Table 2.2), as the parental interviews would suggest. Specifically, at 2 months of age, the timing of the Dutch infants’ excursions outside the house (and thus presumably, their exposure to direct sunlight) was more regular, that is, was significantly more likely to occur at the same time on the 2 days studied. This contrast holds at 6 months, and has in fact grown larger (though with greater variation, hence marginal *p*‐value).

**Table 2.2:** Observed Regularity

Comparison of Day 1 and Day 2	NL	US	Statistic	*p*
2 Months: Outside (percent same)	0.17	0.08	*z* = 2.19	.03
2 Months: Sleep (percent same)	0.74	0.65	*z* = 2.88	.004
2 Months: Feeding bouts				
Difference in number of bouts	1.20	1.12	*t* = 0.35	.72
Correlation of number of bouts	0.49	0.71	*z* = −1.18	.12
6 Months: Outside (percent same)	0.19	0.04	*z* = 1.70	.09
6 Months: Sleep (percent same)	0.81	0.76	*z* = 1.74	.08
6 Months: Feeding bouts				
Difference in number of bouts	0.08	1.40	*t* = −2.05	.04
Correlation of number of bouts	0.78	0.42	*z* = 1.88	.03

With regard to feeding times, it can be seen that at 2 months, there is no significant difference in the daily variation in the number of feeding bouts, but by 6 months, the Dutch mother‐infant pairs are significantly more regular in the number of feeding bouts per day. In addition, individual differences within the sample are then more stable in the Netherlands, that is, the day‐to‐day correlation is higher than in the US (at 2 months, the difference in correlations is not significant, though curiously in the opposite direction).

As indicated above, the U.S. mothers mentioned a great diversity of activities in the context of scheduling: playing, snuggling, videos, change in caretakers, and more. It proved difficult to reliably mark these events in the diary entries, however, so we have not been able to assess the actual regularity in these events.

Finally, with regard to what is perhaps the main point from the mothers’ perspective, the Dutch infants are strongly more regular in their sleep at 2 months. By 6 months, both groups have become more regular in the timing of their sleep, and the group difference has shrunk and is only marginally significant.

## Discussion and Conclusions

The findings presented here indicate that, consistent with prior research (Harkness et al., 2007; Super et al., 1996), regularity in infant care is a more salient concept in this community‐based sample of Dutch mothers than it appears to be for the U.S. mothers of northeast Connecticut. The present report goes beyond a comparison of beliefs to indicate the maternal practices that mediate between those beliefs and developmental outcomes in the two communities. Specifically, the Dutch mothers in this study instantiated their beliefs in practices aimed at establishing and maintaining regularity in the diurnal rhythm through four types of zeitgebers—sleep, feeding, time spent outside, and other activities. Moreover, in actual observed occurrences of regularity in sleep, feeding, and time spent outside, a more stable diurnal rhythm is achieved among the Dutch infants. The Dutch mothers in this study reported putting their belief in regularity into action by enacting an array of strategies to foster it, such as shaping the baby's sleep pattern, “stretching” the time between feedings, engaging in soothing practices to help the baby go to sleep, and adhering to regular times to be outdoors. In addition, they were more likely than the U.S. mothers to express a belief that caregivers play an important role in guiding the baby toward a restful, regular, healthy daily routine. They seemed comfortable with the idea that even young babies can be expected to adjust their own schedule to external demands, such as the anticipated schedule of daycare and the needs of other family members including themselves. Additional evidence of the Dutch mothers’ awareness of regularity as an organizing principle can be found in their more frequent references to precise clock times, preceding events, and general parts of the day in describing their infant's daily routines. In fact, observed diurnal rhythms of the infants show regular occurrences of both sleeping and spending time outside already at 2 months old. The 6‐month‐old Dutch infants show a more stable rhythm in their daily number of feeding bouts.

In contrast, the U.S. mothers generally saw themselves as playing a much less active role in guiding their baby toward a regular routine. Although they often indicated they hoped their infant would develop a routine sooner rather than later, particularly with regard to sleep, they were less likely to feel that they could—or even should—enact strategies to establish a regular schedule in the early months. Keenly observing the baby's patterns as they emerged, these mothers tended to construct their own days accordingly. The one culturally specific sleep‐related strategy that they reported, that of taking the baby into their own bed for at least part of the night, is distinctive in that it signifies, again, an accommodation to the perceived needs of the baby. This less active role of entraining regularity is also visible in the diurnal rhythms of the U.S. infants, which show less regular occurrences of sleeping, feeding, or spending time outside.

These results provide a well‐articulated example of two distinctive cultural models that are systematically, if sometimes implicitly, related to other cultural models about the child, the family, and the self as parent. Furthermore, these two cultural models exemplify the motivational qualities, or “directive force,” of cultural models related to the self (D'Andrade & Strauss, 1992). For the Dutch mothers in our study, it seems clear that establishing a regular schedule of sleep, eating, and time outside was a highly salient concept—in Levy's terms (Levy, 1973; Wu & Dunning, 2018), a “hypercognized” idea—that was linked to other ideas about the baby's development related to rest, emotional stability, and even love. Successfully adhering to a regular schedule, for these mothers, was a mark of parental competence—even if the baby did not always fully cooperate. In contrast, the U.S. mothers had much less to say about the theme of regularity, and when they did talk about it, they tended to emphasize its contingent qualities or even to assert that they thought regular schedules should *not* be imposed on babies in the first 6 months of life. For these mothers, talk about regularity was not a compelling topic in and of itself, but it gained motivational force through its connection to culturally shared ideas about the baby's development as autonomously driven rather than shaped by parenting practices. Being a “good mother,” in this context, is marked by being able to follow the baby's cues for sleeping and eating, even at the expense of their own need for rest and time to attend to other things.

As LeVine and colleagues (LeVine et al., 1994) have pointed out, there is often a close connection between culturally shared “folk psychology” and scientifically based “empirical psychology,” and the same could be said of culturally variable pediatric advice about best practices for parenting. Although “the three R's” originated from the early 1900s mostly as an approach to increase infant health and hygiene, the concepts of rest and regularity are still espoused by parents and professionals. The core ideas, in fact, are often mentioned in documents published by the national well‐baby clinics (part of the National Health Service, providing postnatal home visits, follow‐up consultations, and comprehensive health care to all mothers and infants in the Netherlands), such as their new maternal guide and on their website (Kesler et al., 2018; Online Consultatiebureau, 2019), and are evident in many Dutch websites giving pediatric and child‐rearing advice (https://www.oudersvannu.nl; https://wij.nl). They are also still salient in much pediatric thought, as indicated by multidisciplinary research and guidelines on interventions for excessive crying (LaHaye et al., 2013; Rijlaarsdam et al., 2016), which recommend regularity, uniformity, and stimulus reduction as the best behavioral intervention, and many of which are referenced by the new Health Service book for mothers. Similarly, with regard to feeding, official advice (Online Consultatiebureau, 2019) indicates that babies need to drink 8–12 times per 24 hours in the first few days and at least six times per 24 hours in the first few weeks; and they also state that after six weeks most parents have found a set feeding schedule. Several Dutch websites and well‐baby clinics recommend feeding schedules (see, e.g., https://www.mamaenzo.nl/voedingsschema-maand-tot-maand/3/). It is thus clear that feeding at regular intervals remains part of the Dutch cultural model for good infant care. To the best of our knowledge, no recent studies have addressed the parenting model of rest and regularity in the Netherlands. However, a recent study on parental beliefs regarding the support of infant motor development in the Netherlands and Israel did confirm the Dutch emphasis on rest during young infancy as Dutch parents were found to mainly stress the importance of rest and lack of stimulation (Van Schaik, Oudgenoeg‐Paz, & Atun‐Einy, 2018).

The lesser emphasis accorded to regularity by the U.S. mothers in our study does not imply they are not interested in their babies’ sleep (or their own). Indeed, in a later study with samples from Italy, Spain, and Korea, as well as The Netherlands and United States, samples comparable to the present ones, there were no group differences in the parents’ substantial concern with the sleep of their 2‐month‐old infants (Harkness et al., 2007). What parent would *not* be concerned about a good nights’ sleep? No other group matched the Dutch, however, in the emphasis on regularity of routines. The U.S. mothers apparently do not see a strong connection between “sleeping through the night” and a regular daily schedule.

Rather than regularity, the U.S. mothers in the present study were more focused on following the baby's cues. This perspective is consistent with advice presented in many popular books and websites. It is noteworthy, though, that overall there seems to be more variability in the types of advice given to U.S. parents, compared to in the Netherlands. Some sources advocate a more structured approach, as evidenced in web articles promoting Dr. Richard Ferber's method of letting babies cry to self‐regulate their sleep (“Is the Ferber method right for your baby (and you)?”, 2017) (https://www.thebump.com/a/ferber-method, 2020). Dr. Harvey Karp, whose book *The Happiest Baby on the Block*, rose to popularity a few years later, advocates flexible sleep routines aided by methods of promoting sleep that mimic the environment of the womb, such as swaddling and soothing sounds (e.g., “white” noise), as well as a “dream feed” in which the baby is woken before parents go to sleep for one last feeding to get through the night (Karp, 2002). Last, there is a strong movement for baby‐led schedules, some based upon the popular attachment parenting model by William and Martha Sears (Dewar, 2017; Lerner, 2019; Sears, Sears, Sears, & Sears, 2013). Experts favoring baby‐led schedules mostly advise parents to closely watch their baby's hunger and sleep cues as each baby has their own unique schedule and babies should be accommodated in their own needs for fluid and energy. Dewar and Sears even discuss scientific evidence of poorer socioemotional or academic outcomes for children who as infants had been subjected to scheduled feedings as opposed to on‐demand feedings (e.g., Iacovou & Sevilla, 2013). In addition to the much larger population and greater cultural diversity in the United States compared to the Netherlands, the greater variety of advice in the United States may also reflect parents' need for ways to manage day‐to‐day (or night‐to‐night) while searching for the often‐ambiguous signals from their baby. If sleep schedules are basically up to the individual infants as they mature, and there is no dominant cultural model of what parents can actually *do*, the way is opened for a variety of beliefs and strategies for short‐term coping (Super and Harkness, 1996).

This cultural model of baby‐led schedules, shared by both pediatric sources and many of the U.S. parents themselves, is further reflected in popular advice to parents that stresses the importance of early stimulation as essential for development—an idea that the Dutch mothers rarely mentioned. U.S. parents are offered an array of books on techniques to “stimulate” infants’ brain development, with titles such as *Bright from the Start*, (Stamm & Spencer, 2007), *How to Have a Smarter Baby* (Ludington‐Hoe & Goland, 1985), and *Baby Prodigy* (Candiano‐Marcus, 2009). There is as well a proliferation of educational videos available through Netflix, YouTube, and various apps, and even lessons in sign language to “boost your baby's brain development” (“www.babysignlanguage.com,” 2019).

Given the clear contrast between not only the Dutch and U.S. mothers’ ethnotheories, practices and stability in young infants’ diurnal rhythms but also pediatric advice in each country, the question arises: What is really best for babies? When parents aim to achieve regular diurnal rhythms early in life, and this belief is instantiated in specific practices to entrain regularity, our study shows that infants’ occurrences of sleep, feeding, and time outside are indeed more regular. In some regards, the benefits of a more regular and restful schedule are obvious. Our earlier research with Dutch and U.S. infants showed that a group of Dutch babies (whose mothers endorsed the same cultural models as did the mothers in the present study) were quieter and less fussy, and their mothers reported fewer sleep problems (Super et al., 1996) than did their U.S. counterparts. In addition, it is evident from the present study that the Dutch mothers felt more entitled to take their own needs and the needs of the rest of the family into consideration than did the U.S. mothers, who seemed convinced that being a “good mother” necessarily entailed prioritizing the baby's perceived needs almost to the exclusion of their own. Yet, few of the U.S. mothers in our study would be likely to state with satisfaction that “every day is the same,” as did some of the Dutch mothers. Just as they perceived their babies as needing higher levels of stimulation and novelty, the U.S. mothers also seemed to desire a more varied and exciting daily routine for themselves, even at the expense of a good night's sleep. Possibly the lesser stability in daily rhythms matches not only the parental beliefs and practices, but also these parents’ desire for a stimulating and varied routine.

The mothers in our two cultural samples were evidently aware of different approaches to getting the baby on a schedule, but in the examples described to us, the local cultural model proved more successful than any alternative. This may be due in part to the fact that in each case, the local ethnotheory was strongly backed by “expert” advice and to some extent by the local ecology. In particular, the regularity in time spent outside is more easily accessible in the Dutch sample, where mothers could generally go out to do errands on foot in the nearby village. The U.S. mothers needed a car to travel the longer distances to shops and friends’ homes. In addition, the streets in the Dutch study villages were laid out with ample sidewalks as well as demarcated bicycle paths, whereas the narrow, often winding roads in the rural and suburban towns where the U.S. mothers lived were not well suited to pedestrians, especially ones pushing baby carriages. Altogether, it seems likely that for establishing a regular schedule in one domain such as eating or sleeping, the rest of the cultural model of the “3 R's” (including plenty of rest and limiting stimulation) has to be put into practice as well. In this regard, the redundancy of cultural models as they are instantiated in the child's developmental niche is a basic attribute that is often overlooked when members of one culture attempt to adopt a custom from another cultural context (Super & Harkness, 1999, 2002).

Although both cultural models regarding regularity have their limitations, it appears that both offer important perspectives on what babies need for healthy development. As the late British nurse Tracy Hogg suggested, imposing a regular schedule in babies’ lives may be a helpful antidote to the current U.S. middle‐class preoccupation with stimulation and responsiveness to the baby's perceived individual needs (Hogg & Blau, 2005). On the other hand, even in the Netherlands, several critical comments can be found regarding set schedules, and current advice for parents in the media recommends regularity of routines as more important than clockwise regularity in feeding and sleeping (e.g., Ouders van nu, 2020; wij, 2020). These critical comments notwithstanding, the current study found clear evidence of the focus on zeitgebers such as sleep, feeding and time outside in the Dutch sample versus social activity in the US sample. While the mature human biological rhythm is largely self‐sustaining (Aschoff & Pohl, 1978; Blumberg et al., 2014; Czeisler et al., 1999), the current study specifies the influence of culturally sustained developmental models on entrainment of the circadian rhythm through zeitgebers in both cultural contexts. However, getting the baby on a schedule seems to be a more urgent theme for the Dutch parents, than for the US parents and the Dutch infants respond to the regularity in zeitgebers. This application of developmental models in practices and their effects on infant daily rhythms matches expert advice in both countries.

In today's “global village,” we now have new opportunities to learn from each other's cultures, to the benefit of all. U.S. mothers might find some useful tips in the practices that Dutch mothers employ to promote the early establishment of diurnal rhythms, especially in the critical domain of sleep. At the same time, Dutch mothers might find it helpful to be reminded that not all babies are equally ready to sleep and eat at set intervals—innate, individual differences do play a role here as in other aspects of development. For parents in the post‐industrial world whose daytime routines are governed by the clock, getting the baby on a schedule is an imperative—but as the present study indicates, there are different paths to reaching that goal within the larger cultural contexts of good parenting.
